# Machine learning modeling and analysis of prognostic hub genes in cervical adenocarcinoma: a multi target therapy for enhancement in immunosurveillance

**DOI:** 10.1007/s12672-025-02834-3

**Published:** 2025-07-13

**Authors:** Madiha Jabeen Abbasi, Rashid Abbasi, ShuPeng Wu, Md Belal Bin Heyat, Ding Xianfeng, Huijie Jia, Aiwen Zheng

**Affiliations:** 1 School of Life Science and Medicine, Zhejiang Sci-Tech University, Hangzhou, 310018 China; 2 College of Computer Science and Artificial Intelligent, Wenzhou University, Wenzhou, China; 3 CenBRAIN Neurotech Center of Excellence, School of Engineering, Westlake University, Hangzhou, 310018 China; 4 College of Notoginseng Medicine, Wenshan University, Wenshan, 663099 Yunnan China; 5 Oakham School, Chapel Close Oakham, Rutland, LE15 6DT UK; 6 Department of Gynecologic Oncology, Zhejiang Cancer Hospital, Hangzhou Institute of Medicine (HIM), Chinese Academy of Sciences, Hangzhou, 310022 Zhejiang China

**Keywords:** Endocervical adenocarcinoma, Molecular docking and dynamics simulation, Hub genes, MTT Assay, Functional enrichment analysis, Tumor immunosurveillance

## Abstract

Endocervical adenocarcinoma (ECA) the fatal and intrusive subtype of cervical carcinoma is on rise from the last decade. Its improper detection leads to worst clinical outcomes that urges the discovery of novel biomarkers. Therefore, we proposed insilico and invitro based approches to identify key genes that could be used as potential targeted therapies. RNA-seq and gene expression data was operated via R-programming that identified 11,592 differential expressed genes which are mainly enriched in metabolic pathways, chemical carcinogenesis-receptor activation, amoebias, MAPK and PI3K-AKT signaling pathway. Clustering modules and hub genes were retrieved to design network of immune cells with varying expression using multiple statistical algorithms. The Drugs targeting hub genes were determined from Drug gene interaction database which was further categorized for docking and dynamics based simulations. Results indicate high binding affinity of Imatinib compound into active pockets of BIRC5 which is confirmed by cell viability lab experiment. Current study demonstrates novel biomarkers and therapeutic drugs for in depth understanding of endocervical carcinogensis.

**Supplementary Information:**

The online version contains supplementary material available at 10.1007/s12672-025-02834-3.

## Introduction

Cervical carcinoma being a major public health issue, continues to rank fourth malignant tumors among women with an annual incidence of around 662,301 and 348,874 deaths throughout the world [1]. Endocervical adenocarcinoma (ECA) is currently second most prevalent cancer after squamous cell carcinoma (SCC) that affects 25% of cervical malignancies with an expanding ratio as compared to Squamous cervical carcinoma (SCC) [2, 3]. It growing cases effect on individual’s lifestyles and bring socioeconomic burden for country [1]. Despite of high throughput technologies, molecular profiling the genetic makeup of some cancers are still mystery. Endocervix, a channel that connects cervix to uterus in women is lined by numerous glandular cells. This same kind of glandular cell that also produces mucus is considered as the progenitor of endocervical adenocarcinoma [4]. ECA can be categorized into HPV associated and unassociated groups. The ECAs that is linked to HPV may morphologically resemble endometrioid or serous carcinomas and they can also exhibit mucinous characteristics sometimes intestinal differentiation. Among the category of ECA that are not linked to HPV include gastric, clear cell, and mesonephric types as well as minimum deviation adenocarcinomas (MDA), which are also called adenomas malignum [5, 6]. Papillomavirus in humans (HPV) is a key contributing factor to cervical malignancies mainly squamous cell carcinoma; therefore, revelation of HPV vaccinations has significantly reduced the prevalence of this cancer. On the other hand, a portion of endocervical adenocarcinomas that are not related to HPV infection are showing more aggressive outcomes [7]. Generally, HPV 16, 18 and 45 are most related to ECA [8]. Various lifestyles factors like Obesity, using oral contraceptives for more than ten years, having several sexual partners, and hormone replacement therapy are also associated with an increased risk of ECA cancer [9]. The clinical features, histological performance and genetic profiles of ECA and SCC mark distinct and significant difference, yet their treatment approaches remain the same, which is why we are seeing worse ECA prognosis than SCC. ECA patients show higher death rate then SCC [10].

For individuals with early-stage cancer, surgery is considered the best therapeutic choice, with an overall survival (OS) of around 50% after 5 years. In contrast, a later diagnosis deteriorates the patient’s health greatly, with an OS of just 20% after chemotherapy and radiation therapy [11, 12]. Most of the time, ECA is discovered at later stages and makes patients ineligible for surgery. So, in order to prevent misinterpretation and to get early diagnosis we need to find therapeutic biomarkers associated with prognosis of ECA.This research offers valuable information on the molecular causes of ECA and helps identify possible biomarkers that were linked to disorders pathways and lead to various malignancies. In this study microarray datasets were collected from UCSC(Xena) tool and Geo database. Protein- Protein interaction among overlapping DEGs were determined by STRING database and its network was drawn by cytoscape. With help of MCODE module analysis cluster analysis was anticipated. CytoHubba is addressed for hub gene identification based on 5 different methods. To discover clinical and prognostic value of each hub gene we used GEPIA2 to get important information regarding gene expression and survival analysis of hub genes in ECA in term of boxplot and KM plot. In order to confirm the levels of protein expression encoded by hub genes, we used immunohistochemistry data from the Human Protein Atlas (HPA, https://www.proteinatlas.org/). By using the TIMER 2.0 database, we explore a link between hub gene expression and the infiltration of immune cells (CD8+ T cells, CD4+ T cells, B cells, dendritic cells, macrophages, and neutrophils) in patients with endocervical adenocarcinoma. For drug -gene network construction we find FDA approved drugs against our hub genes by DGidb tool then imatinib compound were docked into active site of BIRC5 after evaluating ADMET (Absorption, distribution, metabolism, excretion) properties of compound. Molecular dynamics simulation was performed after docking to determine stability of compound then MTT experiment is designed in laboratory to measure drug sensitivity and activity in human bodies. This study provided new guidance for further research on ECA. It explored better pathways for diagnosis and prognosis endocervical adenocarcinoma patients.

## Related work

Ojesina at el. [13] have worked on 24 ECA patients to determine genetic alterations within whole genome sequence of cancerous tissues. Author performed exon sequencing using Illumina Hiseq and then D-toxoG for mutation filtration among sequencing data. Hierarchical clustering was also explored by heatmap and ggplot. Then his work is followed by gene expression interpretation by Cufflinks and Cuffdiff, HPV typing by PCR and mass spectroscopy and HPV integration location by Path-seq. Author find out given genes with their alteration percentage TP53(9%), ELF3 (13%), CBFB (8%), PIK3 CA (16%) and KRAS (6%) [13]. However, his work was more confined to squamous cervical malignancy than endocervical adenocarcinoma.

Alexi et al. [14] find out genomic alteration between cervical adenocarcinoma and squamous carcinoma. Author collected samples and extracted and quantified about 250 nanogram DNA from samples. Then HPV F-PCR was used for HPV genotyping. Mutation was detected by Oncomap high-throughput genotyping platform and 1250 alteration were integrated with 139 mutated genes. Immunohistochemistry was also performed by author then all the results were statistically validated by Fish exact test between genomics mutations and other variables such as type and grade of tumor, HPV genotype of cancerous tissues etc. According to results HPV 18 were 7.7% present in cervical adenocarcinoma which are more common than squamous cervical cancer. Furthermore, author find ECA comes out most common in young women then older ones. Mutated genes come out by this work in ECA was PIK3 CA (31.3%), KRAS (8.8%) and SKT11(5%) in ECA samples while EGFR mutation was only present in squamous cancerous samples [14].

Ding et al. [15] worked to find biomarkers associated with cervical malignancies. Author retrieved transcriptomics data and their annotations from publicly freely available TCGA database and GENCODE project respectively. Author used CESC (Cervical adenocarcinoma and squamous carcinoma) data. Then edgeR package from Rstudio is used for normalization and quality control of data and differential expressed genes based on these 2 parameters (log twofold changej ≥ 2 and (P-value less than 0.05) were generated. Hub nodes and network were constructed by WGCNA R package. PPI network among differential genes were analyzed by STRING database and clusters were construed from MCODE module of Cytoscape software. Author used DAVID for GO and KEGG enrichment analysis then validate protein expression by HPA database between normal and tumor tissue. Enriched GO terms come out from this research is extracellular exosome while KEGG enriched pathways were cytokine-cytokine receptor interaction and Toll like receptor signaling pathway. The total 144 nodes along with 285 edges were noticed in PPI network of DEGs. Survival analysis shows that following DEGs (DES, C9orf84, TCEAL2, FOXP3, IL21R, PDE2 A, IRF6, HCAR3, CXCL9, IFI30 MYH11, ADGRF4 and ANXA8L1) were associated with prognosis of cancer while some are negatively co-related with survival analysis among which PDE2 A, HCAR3, IRF6, ADGRHF4 and ANXA8L1 genes are involved. Author also used HPA database for protein level expression confirmation between normal and tumor tissues although pathological data for DEmRNA were not available at HPA but he found PDE2 A expression in tumor tissue were elevated than normal cervical tissues. Hence PDE2 A was a prognostic biomarker used for endocervical and squamous cervical carcinoma according to results [15].

However current study is better because it involves more comprehensive data analysis on ECA patients. Epidemiology of mutation with their sites were mentioned along targeted drugs for ECA in the present studies. For validation of gene and protein expression between tumor and normal tissues GEPIA2 and HPA database has used. It gives more insightful directions for future research.

## Material and methods

### Machine learning pipeline

Machine learning and bio-informatics has revolutionized the research by modern cutting-edge technologies. Artificial intelligence has upgraded the emergence of pharmaceutical drug technology in recent years. Computer aided drug designing is followed by multiple steps including designing, administration and clinical analysis with use of ML techniques. ML have addressed several exponential biomedical data related with clinical life sciences by different tools and approaches. We assembled ML pipeline in TCGA-CESC and GEO datasets by R and Python language. Data normalization and prepossessing were done to alleviate class imbalance by synthetic Minority Over Sampling Technique (SMOTE). Normalized features were anticipated by MinMax scaler by scikit-learn [16, 17]. For CESC patients’ different features including patient's age, gender and stages of disease were calculated from their expression level. We used 15 algorithms to predict the best model for identification. The model resilience was evaluated by stratified tenfold cross validation. The sketch of whole experimental techniques that underpin this research is given in (Supplementary file. 1).

### Differential gene expression and enrichment analysis

Xena differential gene expression pipeline is used for further analysis of GDC(CESC) data. The normalization procedure (Z normalization) is used to remove variables that can influence the results and transform read counts into useful measurement of gene expression. Principle component analysis is applied for detecting global patterns in high dimensional database. Then Limma voom is used to get differential gene expression (DGEs). For GSE145372 and GSE105409 we used GEO2R tool that contains numerous R packages from Bioconductor based on R programming language. In this study, we applied GEO2R by using GEO query and limma that is used to get DEGs among microarray data. GEO2R is also responsible for quantile normalization of data which ensures value distribution for each of sample must remain identical. SR plot is used to draw volcano plot of DEGS for all three datasets to get graphing and data visualization features together. Then Venny2.1 (https://csbg.cnb.csic.es/BioinfoGP/venny.html) is used to compare three datasets and find out common DEGs among all datasets. To get full annotation of DEGs in term of its biological process, cellular component and molecular function we conduct Gene ontology (GO) with use of David database. For KEGG analysis, we used ShinyGo Webtool to explore its enrichment pathway analysis.

### Protein -protein interaction

To get knowledge about protein -protein interaction among common DEGs of all three datasets we imported our data to Stringv.12.0 database for constructing PPI network. Highest confidence (0.900) was selected for interaction scores among genes so as to get reliable strength of interaction. Disconnected nodes were removed from network by using advanced setting option. Then we used Cytoscape through which we analyzed PPI network among genes and fix some algorithmic parameters by a few computational settings. Then Hub genes were screened by utilizing cytohubba analyzer which is one of package in Cytoscape software. In app manager we had installed this package then we had calculated nodes with highest degree of interaction by computing 5 different methods including Degree, Closeness, Maximum Neighborhood Component (MNC), Maximal Clique Centrality (MCC) and Bottle neck. Top 10 hub genes on basis of its score were filtered and used in further analysis.

### Prognostic analysis of hub genes

GEPIA2 is a valuable web application that integrates extensive in-depth expression analysis and profiling across normal and malignant data. To discover clinical and prognostic value of each hub gene we used GEPIA2 to get important information regarding gene expression and survival analysis of hub genes in ECA in term of boxplot and KM plot.

### Immunohistochemical analysis and mutation prediction

We examined the incidence and kind of Hub gene mutations as well as how these mutations affected patient outcomes using the cBioPortal (http://cbioportal.org). Which is one of an open-access resource for examining, displaying, and interpreting multi-dimensional cancer genomic data [18]. Methylation data between normal and tumor samples were also accessed from this website to understand progression and epigenetic regulation of cancer cells. Then Tracking tumor immunophenotype (TIP) database is employed to calculate seven step immune cycle data across CESC samples by demonstration of (step1) antigen recognition, (step 2) antigen presentation, (Step 3) activation, (step 4) trafficking of immune cells to tumors, (Step 5) infiltration level analysis into tumors, (Step 6) T cells activation to cancerous cells, (Step 7) killing malignant cells. Correlation analysis using gene expression data and immune TIP score were carried on RStudio.

In order to confirm the levels of protein expression encoded by hub genes, we used immunohistochemistry data from the Human Protein Atlas (HPA) https://www.proteinatlas.org/). The HPA is a database that includes information on immunofluorescence localization that incorporates transcriptomic and proteomic data and covers around 20 prevalent malignancies data.

### Tumor immune infiltration analysis

Within the TIMER database [19], we can find 10,897 different type of tissue samples reflecting 32 distinct cancers from TCGA database. The CIBERSORT deconvolution algorithm was assessed to evaluate immune cell fraction in hub genes. Then Wilcoxon test determined the differential proportion of CD8+ T cells, neutrophils, macrophages, and B cells between mutant and wild type CESC samples. The whole results were analyzed with Spearman correlation where *P*-values < 0.05 were set as statistically significant.

### Statistical analysis of hub genes

To facilitate knowledge about immunooncology TSIDB website is accessed for analyzing association between immune modulators, immune suppressive, chemokines and human leukocyte antigen (HLA) expression between the high and low expression groups of hub genes. Then we calculated microbiome abundance between normal and tumor group of hub genes. Microbial data is retrieved from The Cancer Microbiome Atlas TCMA database (https://tcma.pratt.duke.edu) and filtered by removing lower abundance and lower frequency data. Statistical test was performed using spearman correlation in RStudio (http://srv00.recas.ba.infn.it/atlas/). After that REDI portal (http://srv00.recas.ba.infn.it/atlas/) is used to get RNA editing sites of hub genes were collected and then statistical analysis through R programming were implemented as per suggestion.

### Drug analysis of hub gene

Agonists or inhibitors drugs that interact with hub genes were screened from DGidb online database [20]. DGidb is responsible to generate drug-gene interaction under different criteria. It contains data from different sources such as DrugBank, ChEMBL, PharmGKB, Ensembl and NCBI Entrez [21]. We used FDA approved parameter in this work for getting better drugs. Drug final list is uploaded to Cytoscape database for visualization and drug -gene network construction.

Among hub genes BIRC5 is selected for futher drug analysis. Its X-ray crystalline structure with PDB id: 1X0X is prepared by addition of polar H +, incorporating missing atoms, removal of water, ligands and hetroatoms then it is subjected to minimization. Active grid box is generated and identified from CASTp tool its structure stability is determined from Ramachandran plot. Similarly drug compound with PubChem CID 5291 is also prepared and minimized. To find out binding affinity of Imatinib with BIRC5 Pyrx virtual screening tool is being utilized. For analyzing nature of docked complex in bio-molecular systems iMOD sever was subjected that efficiently work on several dynamics tools like Monte-carlo simulation, vibrational animations, morphing trajectories by calculating Normal mode analysis NMA vector, affine model and arrows field in internal coordinates of BIRC5 complex[22].

### Experimental design (MTT assay)

In current study, ECA cells dosed with imatinib were cultured and poured in 96 well plate at density of 1.5 × 103 for 37 °C. Cells were placed in 5% CO2 incubator for 24 h. After 1 day, 20 µL MTT reagent is loaded to cell plates at 37 °C for 4 h. Followed by time, medium is removed and dimethyl sulfoxide DMSO is added to each well and then incubated again for 2 h. Then absorbance is monitored at 570 nm by microplate reader. The whole reaction is prevented by sunlight by placing it in dark region.

## Results

**Fig. 1:**
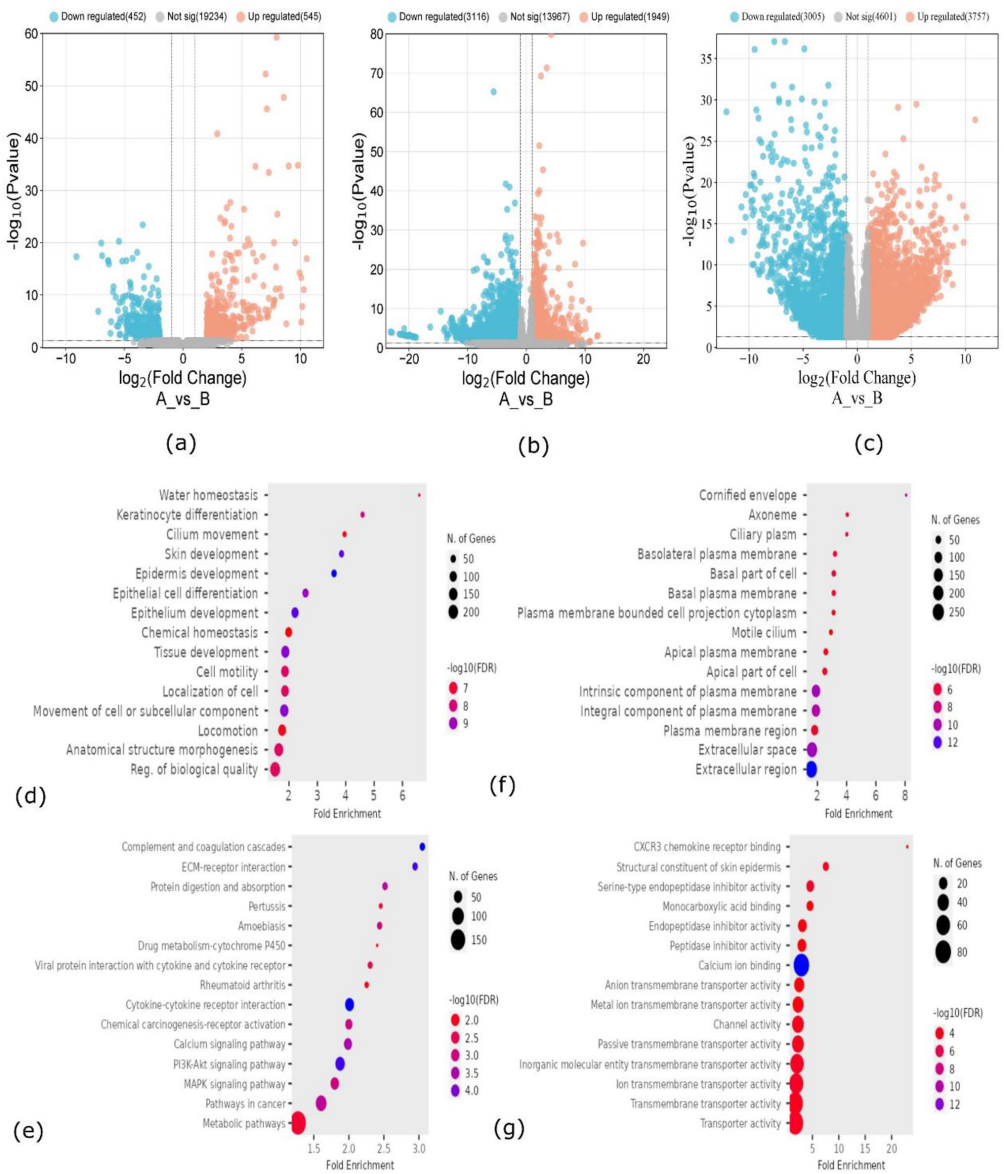
represents volcano plot designed through Sr-plot software. **a** TCGA data, **b** GSE145327, **c** GSE168244 where orange dots represent up regulated and blue dots represent down regulated while gray one represents non-significant genes. On x-axis there is fold change value and y -axis we have p-value. **d** biological process, **e** Cellular component, **f** Molecular function, **g** KEGG pathways. The number of genes is shown on the x-axis, while the keywords are shown on the y-axis

We executed ECA expression data from three different datasets that are GDC TCGA (CESC), GSE145372 and GSE168244 respectively. For DEGs among these datasets we set off a standard in terms of P-value and Log2 FC value in order to get significant genes related with endocervical adenocarcinoma. The P- value for all DEGs must be less than or equal to 0.05 and Log2 FC value must be greater or equal then 2 for up regulated genes. In this way we retrieved 11,592 DEGS among which GSE145372 datasets have 613 up regulated genes and 544 down-regulated genes, similarly GSE168244 contains about 903 up-regulated genes and 3917 down regulated genes. There are 3246 up-regulated and 2369 down-regulated genes were identified in TCGA (CESC) data as shown in Fig. 1a, b, c. Additional analysis was performed using Venny2.1 software to get overlapping DEGs among all datasets. Venn diagram shows there are 248 common genes between all datasets as shown in Fig. 1d. Enrichment analysis shows that DEGs were enhanced in epithelial cell differentiation, water homeostasis, cilium development and keratinocyte differentiation etc. The molecular function associated with DEGs are enriched in transporter activity, calcium ion binding, monocarboxylic acid binding, CXCR3 chemokine receptor binding and serine type endopeptidase inhibitor activity shown in Fig. 1e. Cellular components involve are cornified envelope, extracellular region, integral component of plasma membrane, apical plasma membrane, basal part of cell and ciliary plasm. The KEGG pathways linked with our DEGs are following as: metabolic pathways, complement and coagulation cascades, chemical carcinogenesis-receptor activation, amoebias, MAPK signaling pathway, ECM-receptor interaction, PI3 K-AKT signaling pathway and calcium signaling pathway can be seen in Fig. 1g.

### PPI network and hub gene identification

**Fig. 2:**
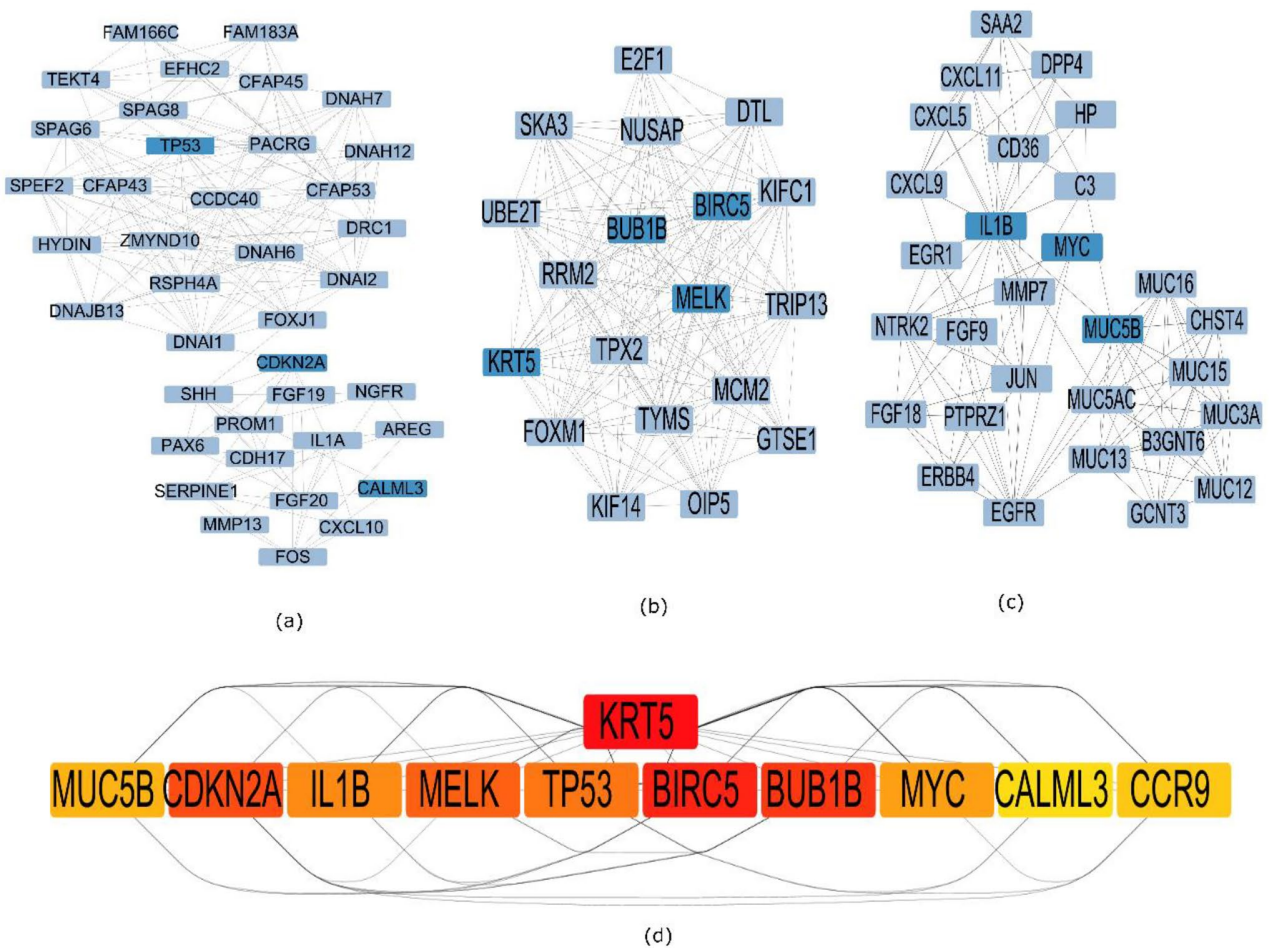
The major module by utilizing the molecular complex detection, **a** (MCODE)Module 1 with a score 17.44(19 nodes,157 edges), **b** Module 2 have a score 19.5(33 nodes,168 edges), **c** Module 3 with a score 6.00 (10 nodes,27 edges), **d** represents rank of identified hub genes by color tone darker red tone goes for higher rank and yellows tone shows lowest rank of hub genes

The Protein–Protein interaction network has 47 nodes, 122 edges, and a PPI enrichment P-value of less than 1.0 as shown in (Supplementary file 2). Three clusters were formed according to the degree of relevance using the MCODE plug-in module. These clusters are shown in Fig. 2a, b and c. In module 1 (a), hub nodes with greater degrees were BUB1B, BIRC5, MELK and KRT5. In module 2 (b), hub nodes with higher degrees were TP53, CDKN2 A and CALML3. In module 3 (c), hub nodes with greater degrees were MUC5B, IL1B, MYC and CCR9.

In addition, five cytoHubba algorithms-Maximum Neighborhood Component (MNC), Maximal Clique Centrality (MCC), Degree, Closeness and Bottle neck-were used to determine which genes in the PPI network served as hubs. Then, ten genes were chosen based on their performance in CytoHubba's five topological methods. After that, hub gene candidates were found by identifying the genes that were shared by all five cytohubba methods. Protein–protein interaction (PPI) network construction shown in (Supplementary file 2).

### Computational validation and survival analysis of hub genes

**Fig. 3:**
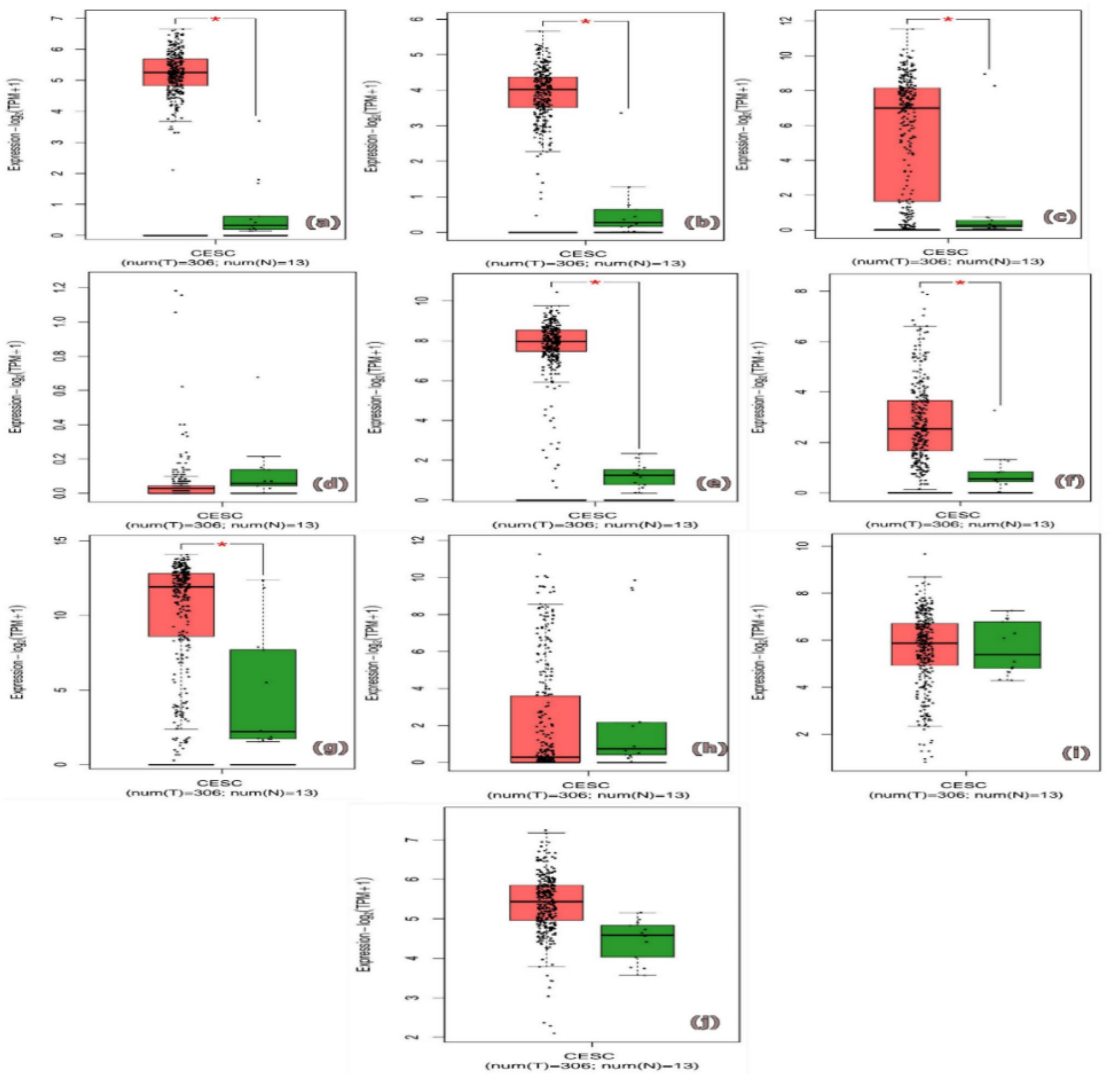
The box plots indicate the expression level of the gene **a** CDKN2 A, **b** KRT5, **c** BUB1B, **d** BIRC5, **e** TP53, **f** IL1B, **g** MYC, (**h**) CCR9, **i** CALML3, **j** MUC5B in GEPIA2 software. The red color represents tumor and green color represent in normal genes

**Fig. 4:**
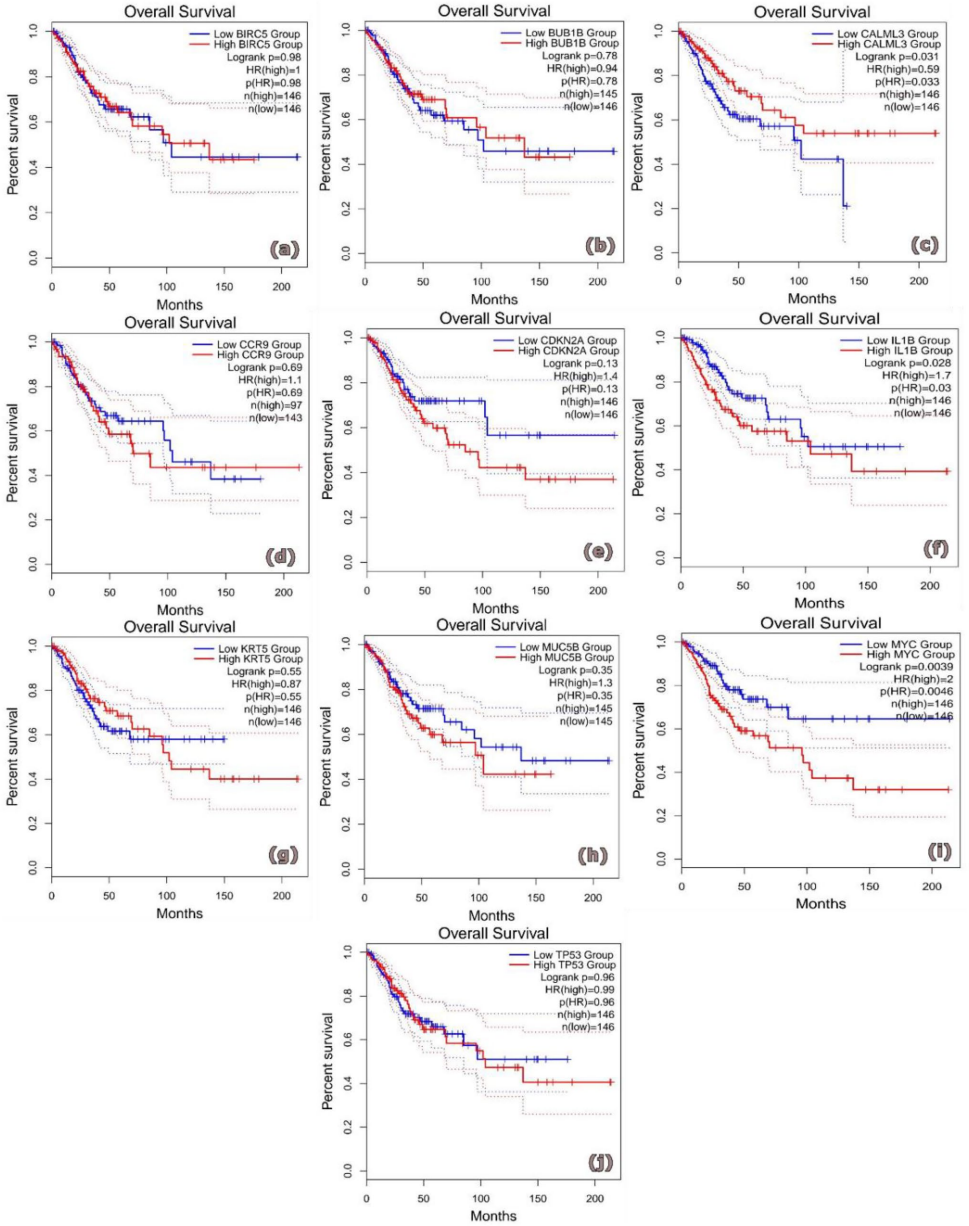
Provides a comprehensive list of the discovered hub genes for ECA along with their prognostic values.HR stands for hazard ratio and ∗ CI for confidence interval. **a** MUC5B, **b** MYC, **c** BIRC5, **d** BUB1B, **e** CDKN2 A, **f** CALML3, **g** CCR9, **h** TP53, **I** KRT5, **j** IL1B

By using the GEPIA databases, we were able to validate that the identified hub genes showed distinct expression patterns in the tumor and non-tumor tissues. The expression of CDKN2 A, KRT5, BUB1B, BIRC5, TP53, IL1B, MYC, and MUC5B mRNA was shown to be up-regulated in tumor tissues when contrasted with non-tumor tissues. The expression of CCR9 and CALML3 mRNA was shown to be considerably reduced in tumor tissues when compared to non-tumor tissues as shown in (Fig. 3) To explore the prognostic importance of the hub genes we received overall survival plots (OS) using Kaplan–Meier method for each candidate hub genes. Our results indicate hub genes clinical significance patients with low gene expression are shown by the black lines, while those with high gene expression are shown by the red lines shown in (Fig. 4).

These findings showed that in ECA patients, increased expression of MUC5B, MYC, BIRC5, BUB1B and CDKN2 A was linked to lower OS rates Furthermore, ECA patients exhibited a shorter OS in correlation with reduced expression of IL1B, CALML3, CCR9, TP53 and KRT5.

### Mutational analysis of hub genes

**Fig. 5:**
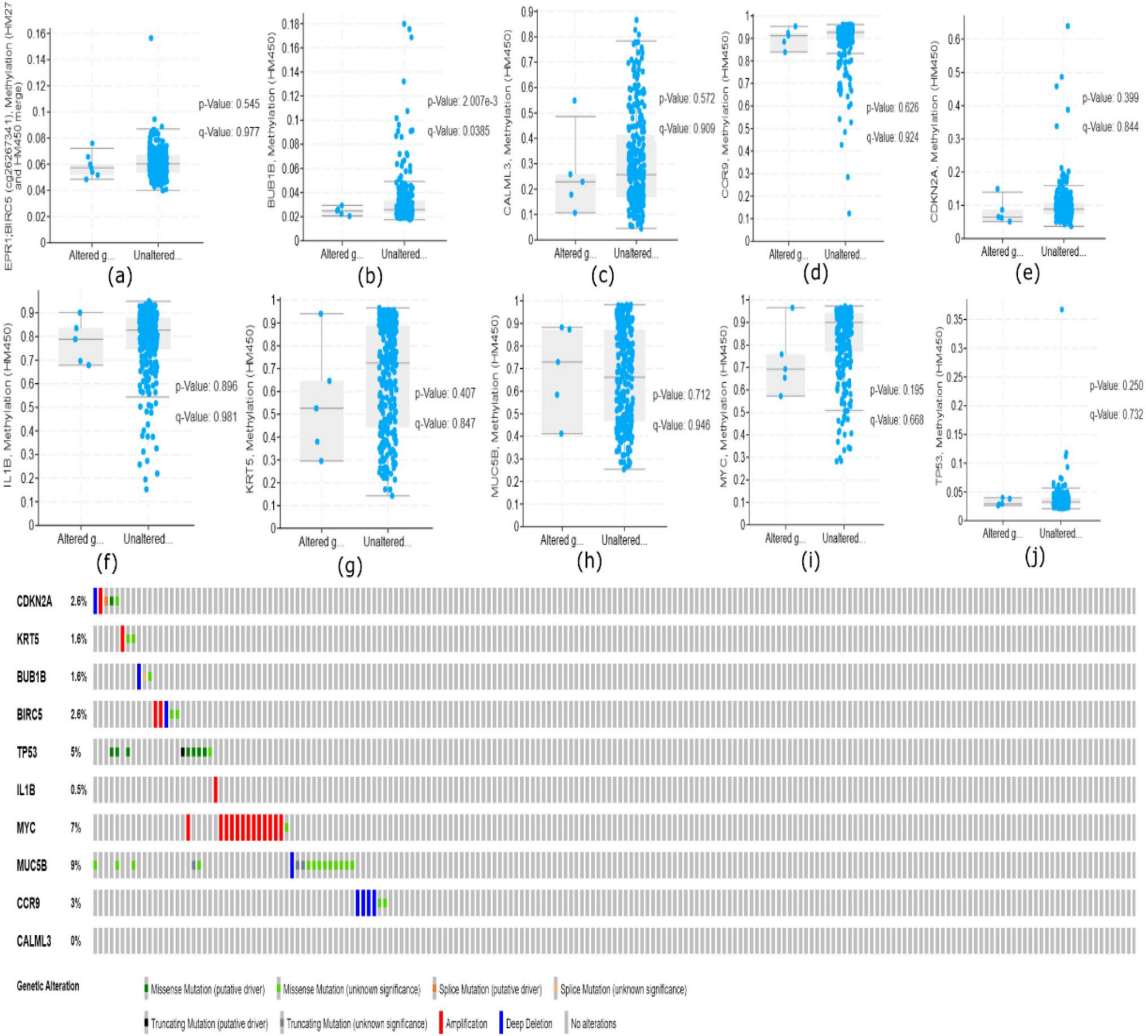
**a** Represents oncogeneic mutation of all hub genes. Figure 4b show methylation between altered and non-altered group of hub genes in CESC. **a** MUC5B, **b** MYC, **c** BIRC5, **d** BUB1B, **e** CDKN2 A, **f** CALML3, **g** CCR9, **h** TP53, **I** KRT5, **j** IL1B

**Fig. 6:**
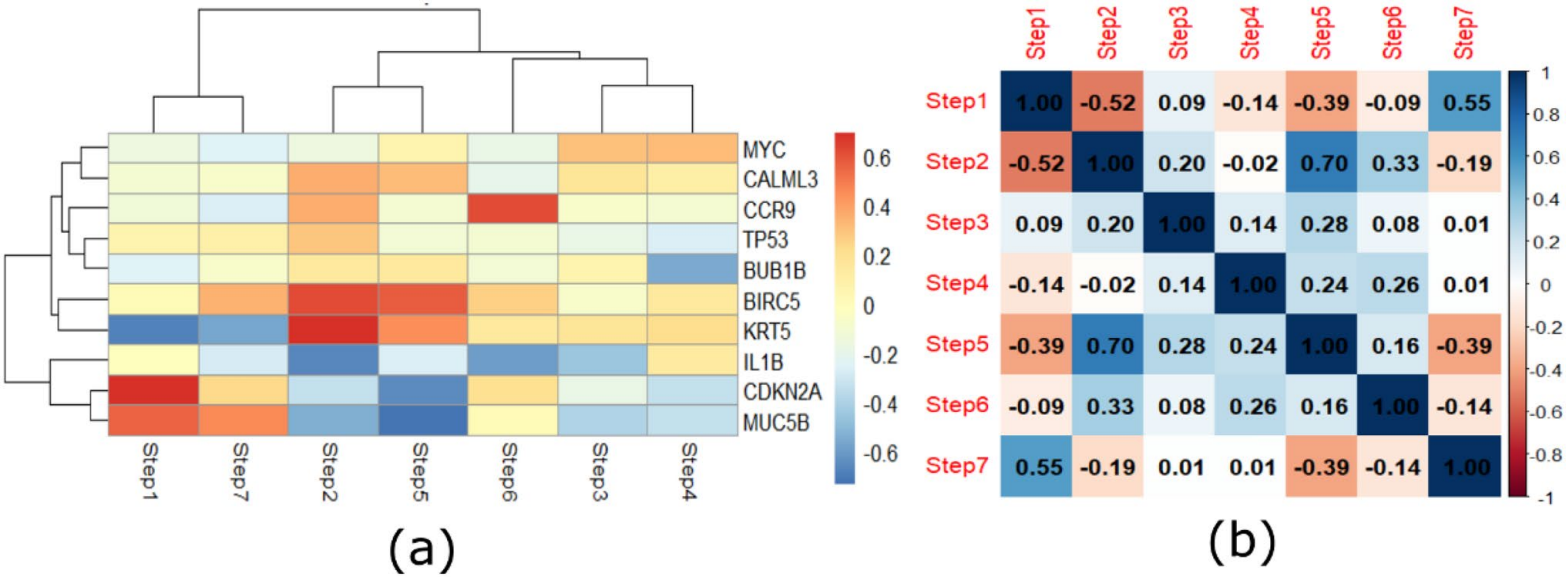
Shows the correlation matrix of TIP score versus hub genes. Red color represents higher positive correlation while blue shows negative correlation between hub genes and tumor immunophenotypes. Range of color represents correlation values

The cBioPortal cancer meta-database was queried for the mutational expression study of ten protein-coding genes. We have queried 10 genes in 191 samples as shown in (Fig. 5) Deep deletion was seen in CCR9, MUC5B, CDKN2 A, BIRC5 and BUB1B genes. While MYC, IL1B, BIRC5 KRT5 and CDKN2 A displayed amplification-mediated increased expression as shown in (Supplementary file 3). Missense mutations were noticed in all genes except CALML3 and IL1B.On the other hand methylation of hub genes were depicted in (Fig. 5a–j) in which CCR9 and MUC5B were hypermethylated in CESC which can lead to gene silencing and promote metastasis of cancer. From tumor immunophenotype analysis we interpreted that higher correlation of CDKN2 A and MUC5B to step 1, BIRC5 to step 2 while CCR9 to step 6 which means their up-regulation is associated with increased antigen recognition, antigen presentation and T cell activation. On the other hand, MUC5B and CDKN2 A is negatively related with step 5, IL1B to step 2 and BUB1B to step 4 which shows up-regulation would cause decrease in infiltration level, trafficking of immune cell and antigen presentation of these genes as shown in (Fig. 6). Lollipop alterations among individual’s hub genes that are CDKN2 A, KRT5, BUB1B, BIRC5, TP53, IL1B, MYC, MUC5B, and CCR9 revealed in (Supplementary file 3).

### Protein expression validation of hub genes

After mutational exploration of hub genes, we examined protein expression level of hub genes. For that validation we used Human Atlas Protein (HPA) database.

**Fig. 7:**
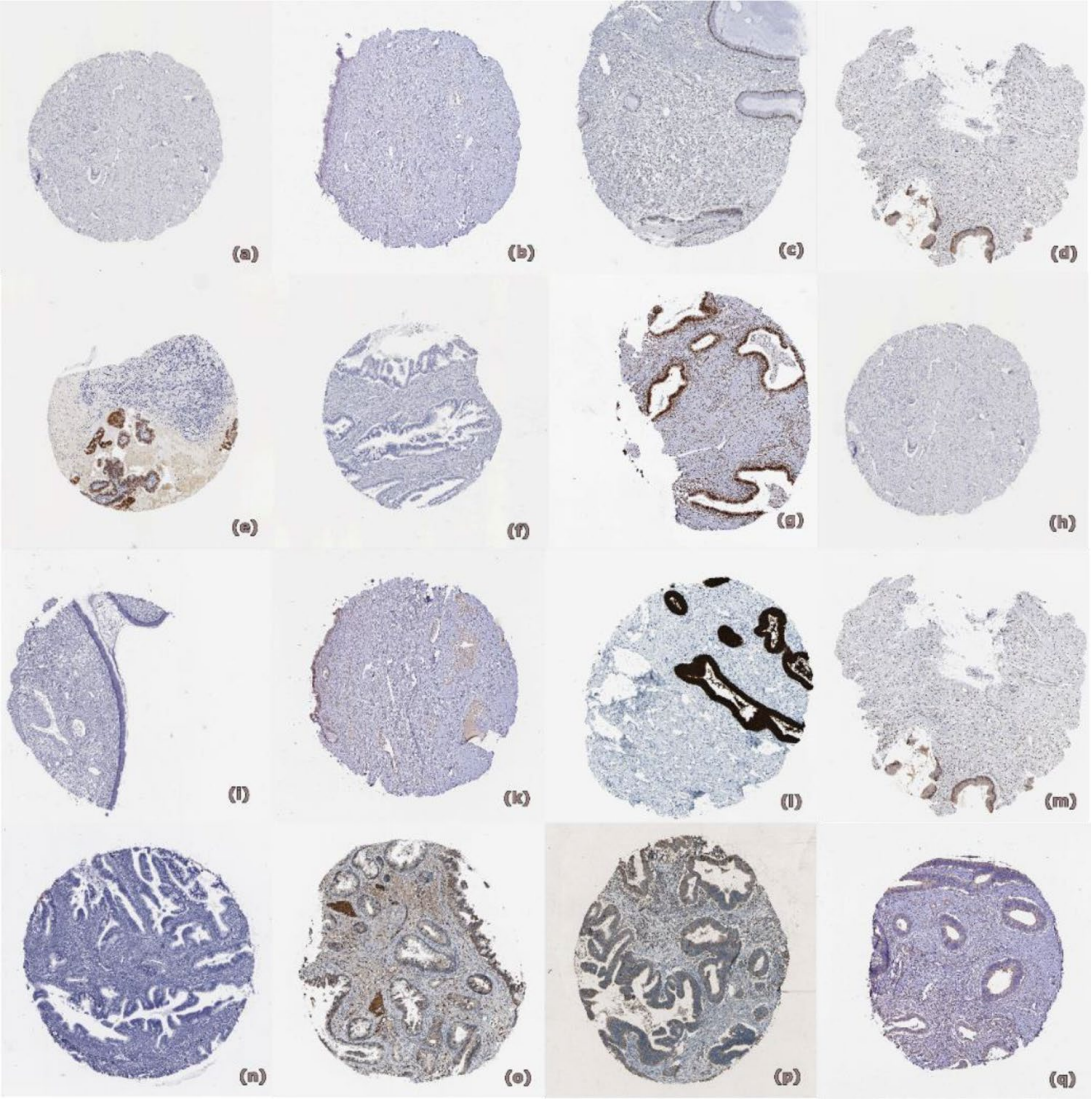
Represents HPA images between normal and tumor tissues where CDKN2 A (**a**) normal and (**b**) tumor, KRT5 (**c**) normal and (**d**) tumor, BUB1B (**e**) normal and (**f**) tumor, BIRC (**g**) normal and (**h**) tumor, TP53 (**i**) normal and (**k**) tumor, MYC (**l**) normal and (**m**) tumor, MUC5B (**n**) normal and (**o**) tumor, CALML3 (**p**) normal and (**q**) tumor

Findings were in agreement with a significant increase in the expression of CDKN2 A, KRT5, BUB1B, BIRC5, TP53, MYC and MUC5B in ECA tissues compared to normal tissues, according to the immunohistochemistry (IHC) staining that was retrieved from the Human Protein Atlas (HPA) database. Also, as shown in (Fig. 7), the expression of CALML3 was noticeably decreased in ECA tissues compared to normal tissues.

### Tumor immune infiltration analysis of hub genes

To determine tumor immune infiltration of CDKN2 A, KRT5, BUB1B, BIRC5, TP53, IL1B, MYC, CCR9, CALML3, MUC5B we used TIMER2.0 database. According to results we received 6 infiltrated tumor immune cells association with each of our hub genes shown in (Table 1). CDKN2 A gene has positive co-relation with all 6 immune cells while BUB1B gene shows negative correlation with B cell, dendritic cell and macrophages and positive co-relation with CD8+ Tcell, CD4+ T cell and neutrophil. The BIRC5 gene have positively associated with macrophages and negatively associated with CD8+ T cell, CD4+ T cell, B cell and neutrophil while it doesn’t have any significant link with dendritic cell. TP553 is also linked positively to CD8+ T cell, CD4+ T cell, B cell and DC while negatively linked with macrophages and neutrophils. ILIB has negative impact on CD8+ Tcell, macrophages and B cell while and MYC has negative effect only on B cell and macrophages. MUC5B is negatively related with CD4+ T cell, dendritic cell, macrophages and neutrophils while CCR9 is negatively associated with only CD4+ T cell. On the other hand, CALML3 is positively associated with all immune cells while KRT5 is negatively associated with B cells and macrophages. Table 2 summarized significant differential composition of immune cells for hub genes across mutant and wild type samples that infiltrate the CESC microenvironment, hence influencing the progression of malignant tumor. Scatter plots of the proportion of immune cells in CESC versus normal samples were plotted in (Supplementary Fig. 4).

**Table 1:** represents hub genes and their associated immune cells. P < 0.05 and P > 0.05

Hub Genes	CD8 + T cell	CD4 + T cell	B cell	Dendritic cell	Macrophages	Neutrophils
CDKN2 A	r = 0.134 P = 2.53e-02	r = 0.225 P = 1.55e-04	r = 0.185 P = 1.99–03	r = 0.176 P = 3.25e-03	r = 0.17 p = 3.61e-04	r = 0.213 P = 3.61e-04
BUB1B	r = 0.153 P = 1.06e-02	r = 0.157 P = 8.95e-03	r = −0.218 P = 2.15e-04	r = −0.126 P = 3.06e-02	r = −0.216 P = 2.91e-04	r = 0.231 P = 1.03e-04
BIRC5	r = −0.135 P = 2.48e-02	r = −0.201 P = 7.51e-04	r = −0.167 P = 5.32e-03	–	r = 0.162 P = 6.83e-03	r = −0.313 P = 1.03e-07
TP53	r = 0.119 P = 4.87e-04	r = 0.137 P = 2.30e-02	r = 0.144 P = 1.62e-02	r = 0.2 P = 8.17e-04	r = −0.184 P = 2.13–03	r = −0.178 P = 2.90e-03
IL1B	r = −0.122 P = 4.33–02	r = 0.168 P = 5.19e-03	r = −0.144 P = 1.68e-02	r = 0.224 P = 1.66e-04	r = −0.14 P = 1.95e-02	r = 0.351 P = 1.81–09
MYC	r = 0.122 P = 4.19e-02	r = 0.238 P = 6.11e-05	r = −0.174 P = 3.62e-03	r = 0.164 P = 6.34e-03	r = −0.132 P = 2.77e-02	r = 0.228 P = 1.33e-04
MUC5B	r = 0.147 P = 1.44e-02	r = −0.15 P = 1.27e-02	r = 0.176 P = 3.29e-03	r = −0.205 P = 6.11e-04	r = −0.236 P = 7.29e-05	r = −0.139 P = 2.08e-02
CCR9	r = 0.194 P = 1.15e-03	r = −0.148 P = 1.36e-02	r = 0.167 P = 5.36e-03	r = 0.166 P = 5.60e-03	r = 0.205 P = 6.00e-04	–
CALML3	r = 0.122 P = 4.27e-02	r = 0.258 P = 1.32e-05	r = 0.134 P = 2.61e-02	r = 0.187 P = 1.77e-03	r = 0.141 P = 1.90e-02	r = 0.178 P = 2.96e-03
KRT5	r = 0.153 P = 1.06e-02	r = 0.338 P = 7.73e-09	r = −0.197 P = 1.01e-03	r = 0.233 P = 9.18e-05	r = −0.154 P = 1.00e-02	r = 0.303 P = 2.62e-07

**Table 2:**
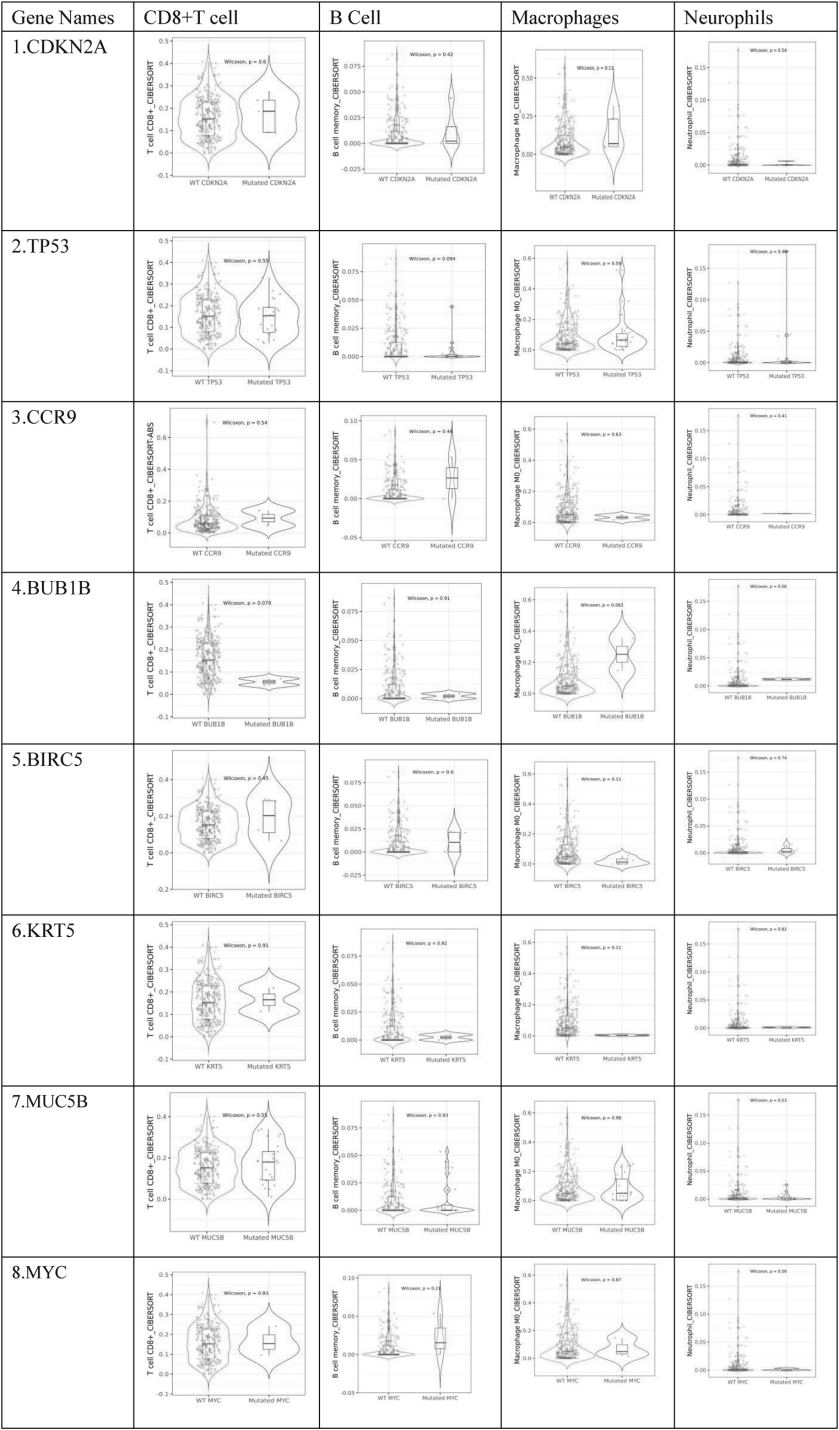
Violin plot between mutant and wild gene samples across immune cells

### Statistical analysis of hub genes

**Fig. 8:**
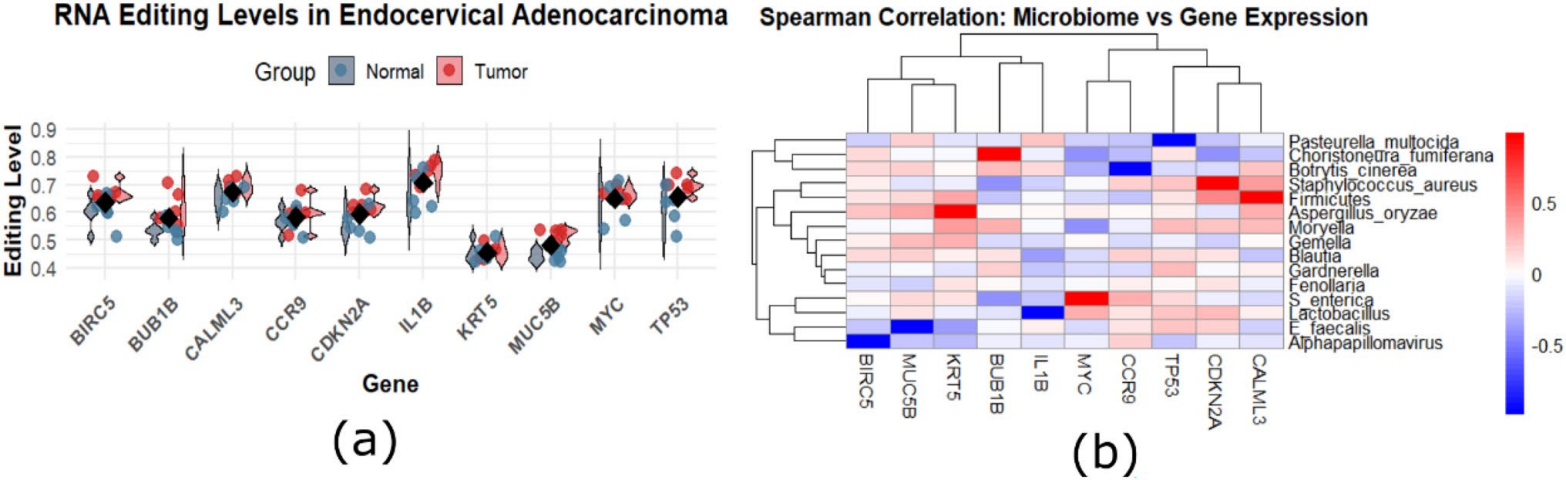
**a** Microbiome abundance of hub genes **b** represents RNA editing analysis of hub genes between normal and malignant genes. Immune markers association to hub genes shown in (Supplementary file 5)

The relationship between hub gene and micro-biome abundance is evaluated by using TCMA database and statistical correlational test in R studio. The correlated matrix with genes on x-axis while micrbial community on y-axis were plotted in which blue cells shows positive correlation while red shows negative correlation shown in (Fig. 8a).The results eloborate that BIRC5 has positive correlation to alphapapillomavirus, MUC5B to E faecalis, IL1B to lactobacillus, TP53 to Pasteurella multocida and CCR9 to bortrytis cinerea. Variation in these microbiome level enhance probility of certain infections which can lead to malignancy. While RNA editing analysis represents higher editing level in cancerous samples as compared to normal ones which can affect cancer related mechanisms as shown in (Fig. 8b). Tumor Immune System Interaction for respective hub genes for different immunomodulators, immunosupressive and chemokines were calculated as shown in (Supplementary file. 5). From results we eloborated that BIRC5 show significant differences (p < 0.05) to PD-L1, IL10, CXCL9, CCL3 while BUB1B to (CTLA4, CXCL10, CCL5, IL6), TP53 to (PD-1, IL6, CXCL10, CCL4, HLA-A), KRT5 to (PDCD1, CXCL9, CCL2), IL1B to (CD274, CXCL10, CCL5), CCR9 to (IFNG, TGFB1, CCL2), CALML3 to (PDCD1, CTLA4, CD8 A), MUC5B to(PD-L1, IL10, CXCL9, CCL3, HLA-DR) and MYC to (PDCD1,CTLA4, CD274, CD8 A).

### Molecular docking and dynamics simulation

**Fig. 9:**
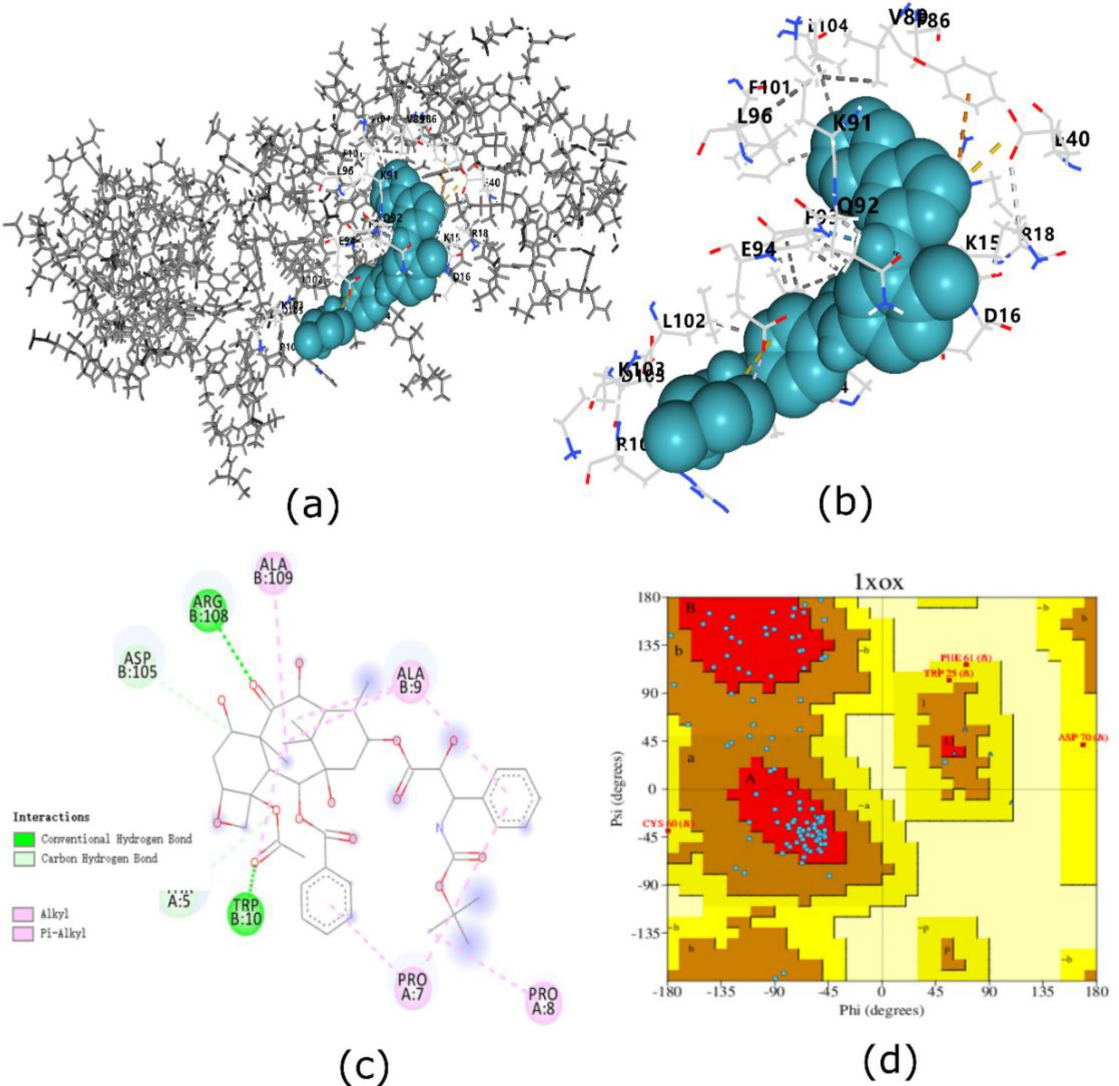
**a** Shows BIRC5 molecular surface image in deep cavity with imatinib attached. Figure 8b represents 3D image while in Fig. 8c dotted lines show interactions in a two-dimensional interaction between a ligand and a protein. Figure 8d represents ramachandran plot of 1XOX

According to results, 271 drugs against 5 hub gene TP53, CDKN2 A, MYC, IL1B and BIRC5 were extracted from DGidb database. All drugs that are approved by Food and Drug administration were selected and matched. Raloxifene hydrochloride, Reveratrol, Genistein, Cisplatin, Temozolomide and Carboplatin drugs were found as common in three genes at same time can be seen in (Supplementary file 6). Melatonin, Lithium, Verapal were found common in MYC and IL1B while Palbociclib, Dabrafenib, Celuximar, Panitumumab, Brigatinib were common in CDKN2 A and TP53. Ibrutnib, Azacitidine, Warfarin, Vironostat targets TP53 as well as MYC on the other hand Imatinib, Indomethacin targets MYC and BIRC5 simultaneously. Raclitaxel and Docetoxel were common in BIRC5 and TP53 genes respectively. From docking studies we revealed stereo conformers of ligand with 1XOX was detected with 9 poses from Pyrx Virtual screening. Among them ligand with minimal energy of 7.5 kcal/mol. As ligand is docked into active site of receptor complex so it defines reliability of our docking. To further evaluate results re docking with same ligand was computed by CB-Dock which gives sames binding orientation of best pose ligand with other cur pockets as shown in (Fig. 9a) Ramachandran plot from PDBsum database shows regions with over 90% of the most favored locations show high-quality model (Fig. 9d). From Imod server dynamics studies revealed stable conformation along various interaction like hydrogen bond and ionic bond between ligand and protein structure. The graphs of simulation in terms of deformability, Bfactor, eigenvalue graph, variance graph and covariance matrix graph can be seen along description in (Supplementary file 7).

Supplementary file 6 shows drugs targeting corresponding genes in which orange circle shows hub genes and blue circle shows drugs.

#### Cell viability assay (MTT assay)

**Fig. 10:**
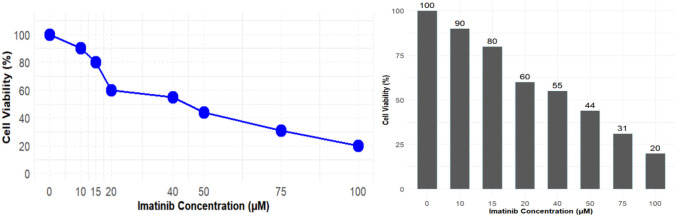
Dose dependent MTT assay impact on cell survival

To validate docking and dynamics simulation of imatinib lab experiment was done to measure cytotoxity of compound. According to results, at control condition the NADPH dependent oxide-reductase an enzyme has converted tetrazolium dye into colored compound which can be visualized. After adding drug compound cell viability shows significant decrease in their concentration which can be seen in (Fig. 10). Drug was added in increasing patterns (10,20,35.50,75,100). At IC50 of 44.55 µM cell shows potent anti-tumor activity. The concentation were reduced at highest dose of drug which indicate potency of compound. At 100 µM cell survival declined at his lowest value which ws below 20%.

## Discussion

From recent decade it was clearly observed that adenocarcinoma of uterine cervix has been rising from 5 to 25% among females [23]. It is treated with surgery following CCRT in early stages. However, exenteration and LEER was performed for advanced stage patients with worst clinical outcomes [24]. Due to improper and poor diagnosis at early stages it become very crucial to discover prognostic biomarker that will help us to understand genetic and molecular makeup of ECA [25]. Therefore, this work demonstrates and validate molecular hub genes by using several tools and databases to explore pathological pathways and drugs associated with ECA. Various research was done in past to demonstrate genetic background of adenocarcinoma patients [13, 26]. However, for this study we collected our data from UCSC Xena and NCBI(GEO) platforms. With the help of GEOR2 and Xena tool 11,592 DEGs related with ECA were screened among which 4762 are up-regulated and 6830 were down-regulated. Common DEGs among three micro array datasets were identified by Venny 2.1 software. The metabolic pathways, complement and coagulation cascades, chemical carcinogenesis-receptor activation, MAPK signaling pathway, ECM-receptor interaction, PI3 K-AKT signaling pathway and calcium signaling pathway are some of enriched KEGG pathways identified in our study. Many studies reveal that higher activation of PI3 K-AKT signaling pathway promote the cervical malignancies. This pathway activated by E6/E7 proteins of HPV which can induce tumorigenesis [27].

Similarly, Ca2+ signaling can regulate different pathways that can activate malignancies [28]. The mutations in MAPK signaling pathways can also mutate Ras and B-Raf pathways that can become a cause of multiple tumours [29]. Previously, many studies revealed that metabolic disorders greatly affect tumor patients and its genetic mechanism [30]. The epithelial cell differentiation, water homeostasis, epidermis development, tissue development and keratinocyte differentiation were some of enriched biological process in our derived DEGs. In papillomavirus replication the epithelial differentiation plays an important role it is part of encoded proteins and regulates the replication process of HPV [31]. HPV was always on top position that can drive major genetic mechanisms in ECA [13]. In multiple researches it was found that Keratinocyte differentiation which can turn on PI3 K/AKT pathway is one of major gene that code protein is present in cervical tissue [32]. Our research has also identified molecular function and cellular component of associated DEGs among which transporter activity, calcium ion binding, monocarboxylic acid binding, CXCR3 chemokine receptor binding, structure constituent of skin epidermis, serine type endopeptidase inhibitor activity was find as enriched MF while cornified envelope, extracellular region, integral component of plasma membrane, apical plasma membrane, basal part of cell and ciliary plasm was enriched cellular components. Chemokines are secreted by tumor cells which can promote cancer proliferation [33]. CXCR3 chemokines can change expression level of some immune cells at cancerous site [34]. On the other hand, calcium ion bind is directly connected with formation and progression of cervical carcinoma. All body cells use calcium ions as signaling molecules to regulate various function. Variation and changes in these ions can lead to the occurrence of cervical malignancies [35]. This study gives us significant protein–protein interaction network among DEGS with 47 nodes and 122 edges from STRING tool. Furthermore, hub genes with highest score were retrieved from five different algorithms of Cytohubba. This study has also provided significant modules from PPI network from MCODE plugin module. We find that CDKN2 A, KRT5, BUB1B, BIRC5, TP53, IL1B, MYC and MUC5B were up regulated genes in ECA while CCR9 and CALML3 were down-regulated. The expression of each hub gene was validated by GEPIA software and its survival analysis information were obtained from Kaplan–Meier plot which shows prognostic importance of these hub genes.

CDKN2 A is highest rank hub gene identified in our studies. Cyclin-dependent kinase inhibitor 2 A (CDKN2 A) is a member of cell cycle regulators it encodes tumor suppressor protein p16. Various cancerous cell from different tumors shows mutation in CDKN2 A. CDKN2 A mutation is linked with various malignancies, including pancreatic tumors, breast cancer, Brain, head/neck and non-melanoma skin cancer [36]. In our work, deep deletion, amplification and missense mutation was identified in ECA patients from CBioPortal tool. A study regarding CDKN2 A mutation shows this gene is 9.77% mutated in endocervical adenocarcinoma patients [37]. Hence, CDKN2 A can serve as a crucial therapeutic agent for better prognosis among ECA patients. TP53 is another significant gene in ECA studies. According to previous work, its altered form is highly expressed in adenocarcinoma patients similar with our research. About 5% missense mutation is found in endocervical adenocarcinoma patients. Tumor suppressant TP53 is crucial for responding against stresses but its genetic alteration is highly concerned with cancer development [38]. KRT5 is another significant hub gene found in our study. This gene is part of keratin family that is present on chromosome 11 [39]. According to previous work, it was revealed that abnormalities in KRT5 is leading cause of many cancers like lung cancer, ovarian cancer and prostate cancer [40]. We find amplification and missense mutation in KRT5 gene from ECA patients with upregulated status. Consistent with previous studies BUB1B is another hub genes whose expression is found higher in ECA from this research. BUB1B is important regulator of kinase which is involved in mitosis. Previous work revealed that, high expression of BUB1B is also associated with many other cancers like breast cancer, gastric adenocarcinoma and hepatocellular carcinoma [41]. BIRC5 gene shows 2.6% mutation major of which are deep deletion and amplification in our research. Survivin protein which is common in cancerous tissues are encoded by BIRC5 gene. This protein works in cell division and it can inhibit apoptosis during early fetal growth [42]. From last few decades, it was showing that this protein expression is elevated in many cancerous cells when compare with normal cells which make it good prognostic marker for cancer [43]. Higher BIRC5 expression is related with worst overall survival in ECA patients.

IL1B an important member of immune system its expression is elevated in cervical malignant patient as compare with normal [44]. Our studies demonstrate amplification in this gene which is related with worst overall survival. CALML3 is another oncogenic gene found in ECA but our studies didn’t find specific mutation from Cbioportal. However, many other studies also suggests that this gene can modulate Wnt/β-catenin pathway in cervical tissues and can promote cancer [45].

Another downregulated gene CCR9 that have deep deletion and missense mutation was point out in this study. CCR9 is responsible for migration and metastasis of many cancer cells [46]. MUC5B and MYC genes were upregulated genes find in current study. Both genes show mutation in ECA expression.

Expression of all 10 hub genes were verified by GEPIA2 and HPA database. According to results, 271 drugs against 5 hub gene TP53, CDKN2 A, MYC, IL1B and BIRC5 were extracted from DGidb database. All drugs that were approved by FDA were selected and matched. Raloxifene hydrochloride, Reveratrol, Genistein, Cisplatin, Temozolomide and Carboplatin drugs were found as common in three genes at same time. Additionally, BIRC5 one of the key gene is further examined for drug designing. Pyrx docking tool was used to determine how BIRC5 binds to Imatinib. The results show that when imatinib interacts with BIRC5, it releases high binding energy of 7.5 kcal/mol. Following molecular docking, MD simulation demonstrates protein’s stable binding to the complex with strong binding affinity. To confirm docking and dynamics clinical experiment cell viability assay was carried on which demonstrate significant decrease in cell viability on addition of imatinib. The concentration of drug was added in increasing patterns (10,20,35.50,75,100). At IC50 of 44.55 µM cell shows potent anti-tumor activity. The concentation were reduced at highest dose of drug which indicate potency of compound. At 100 µM cell survival declined at his lowest value which was below 20%. Microbiome abundance correlation shows significant differentiation in microbial community between normal and tumor samples. For example, Enterococcus faecalis abundance cause vaginitis which can promote malignancy while increased level of pasteurella multocida is also linked with infections and inflammation in CESC genes (44).

## Conclusion

In a summary, a comprehensive computational pipeline is used to explore potential biomarkers associated with development and progression of ECA. The study provides a significant DEGs and their associated pathways which promotes ECA to get better prognosis and diagnosis. In this paper we found that CDKN2 A, KRT5, BUB1B, BIRC5, TP53, IL1B, MYC, and MUC5 were upregulated via CCR9 and CALML3 are downregulated in ECA patients. This integrated investigation has given new insights about biological exploration, progression and pathogenesis about ECA. Nevertheless, further clinical trials to understand deep molecular mechanism of endocervical carcinogenesis is required in future.

### Limitations of study

In current studies high throughput screening, bioinformatics algorithms and chemoinformatic techniques offered thorough understanding of therapeutic hub genes in ECA. However, a real-world trial to predict accurate and deep mechanism of these biomarkers in ECA is required. Due to complexity of human body each drug and biomarkers need evaluation and experimentation which can predict reliability of biological system according to them.

## Supplementary Information

Supplementary material 1

Supplementary material 2

Supplementary material 3

Supplementary material 4

Supplementary material 5

Supplementary material 6

Supplementary material 7

## Data Availability

The datasets generated during and/or analysed during the current study are available from the corresponding author on reasonable request.

## Abbreviations

BP: Biological process
CC: Cellular component
DAVID: Database for Annotation Visualization and Integrated Discovery
DEGs: Differentially expressed genes
DGIdb: Drug-gene interaction database
OS: Survival analysis
FDA: Food and drug administration
GEO: Gene Expression Omnibus
GEPIA2: Gene Expression Profiling Interactive Analysis
GO: Gene ontology
KEGG: Kyoto Encyclopedia of Genes and Genomes
MCODE: Molecular complex detection
MF: Molecular function
ECA: Endocervical adenocarcinoma
PPI: Protein–protein interaction
STRING: Search Tool for the Retrieval of Interacting Genes/Proteins
TCGA: The Cancer Genome Atlas
