# Supplementary material for: Machine learning modeling and analysis of prognostic hub genes in cervical adenocarcinoma: a multi target therapy for enhancement in immunosurveillance
Source: Discov Oncol. 2025 Jul 13;16:1326. doi: 10.1007/s12672-025-02834-3 (PMC12256379; doi:10.1007/s12672-025-02834-3)
Supplement: Supplementary file 2 — Supplementary material 2 [file 12672_2025_2834_MOESM2_ESM.docx]

**Methodology Diagram :**

Endocervical Adenocarcinoma NCBI(GEO database) Differential gene Functional Enrichment PPI Network

Gene expression data XENA (UCSC)TCGA identification(DEG’s) Analysis of DEG’s

Hub Gene CytoHubba Analyzer By Cytoscape MCODE module

identification Clustering

**CDKN2A TP53 KRT5 BUB1B CCR9 BIRC5 IL1B MYC MUC5B CALML3**

Predict Mutational Microbiome Abundance Differential RNA editing Evaluate Gene exp Frequency by CBioPortal of hub genes level for normal and Survival analysis

and tumor samples of hub genes

**PROTEIN-DRUG INTERACTION**

Immunophenotype Analysis EVALUATION OF TUMOR

Molecular Docking of BIRC5 Immune infiltration analysis MICROENVIRONMENT

And Imatinib Differential Immune composition BETWEEN NORMAL AND

Molecular Dynamics Simulation for variants of hub gene TUMOR SAMPLES

ADMET Evaluation of Immune system interaction

Drug Compound Using immune stimulatory,

Suprresive genes, Chemokines etc

**Wet Lab Experimental Validation**  Drug Sensitivity Analysis

**CELL VIABILITY ASSAY /MTT ASSAY**
