# Supplementary material for: Machine learning modeling and analysis of prognostic hub genes in cervical adenocarcinoma: a multi target therapy for enhancement in immunosurveillance
Source: Discov Oncol. 2025 Jul 13;16:1326. doi: 10.1007/s12672-025-02834-3 (PMC12256379; doi:10.1007/s12672-025-02834-3)
Supplement: Supplementary file 3 — Supplementary material 3 [file 12672_2025_2834_MOESM3_ESM.docx]

**Protein -Protein Interaction network：**

With the help of Cytoscape, we looked at PPI networks that had score over 0.900 while removing any disconnected nodes from that interaction. In the network, upregulated DEGs constituted the great majority of the nodes. Genes that are upregulated are shown in red, whereas genes that are downregulated are displayed in blue. Genes are represented by nodes, protein interactions between genes are shown by lines, and the content inside the PPI is represented by network。

**
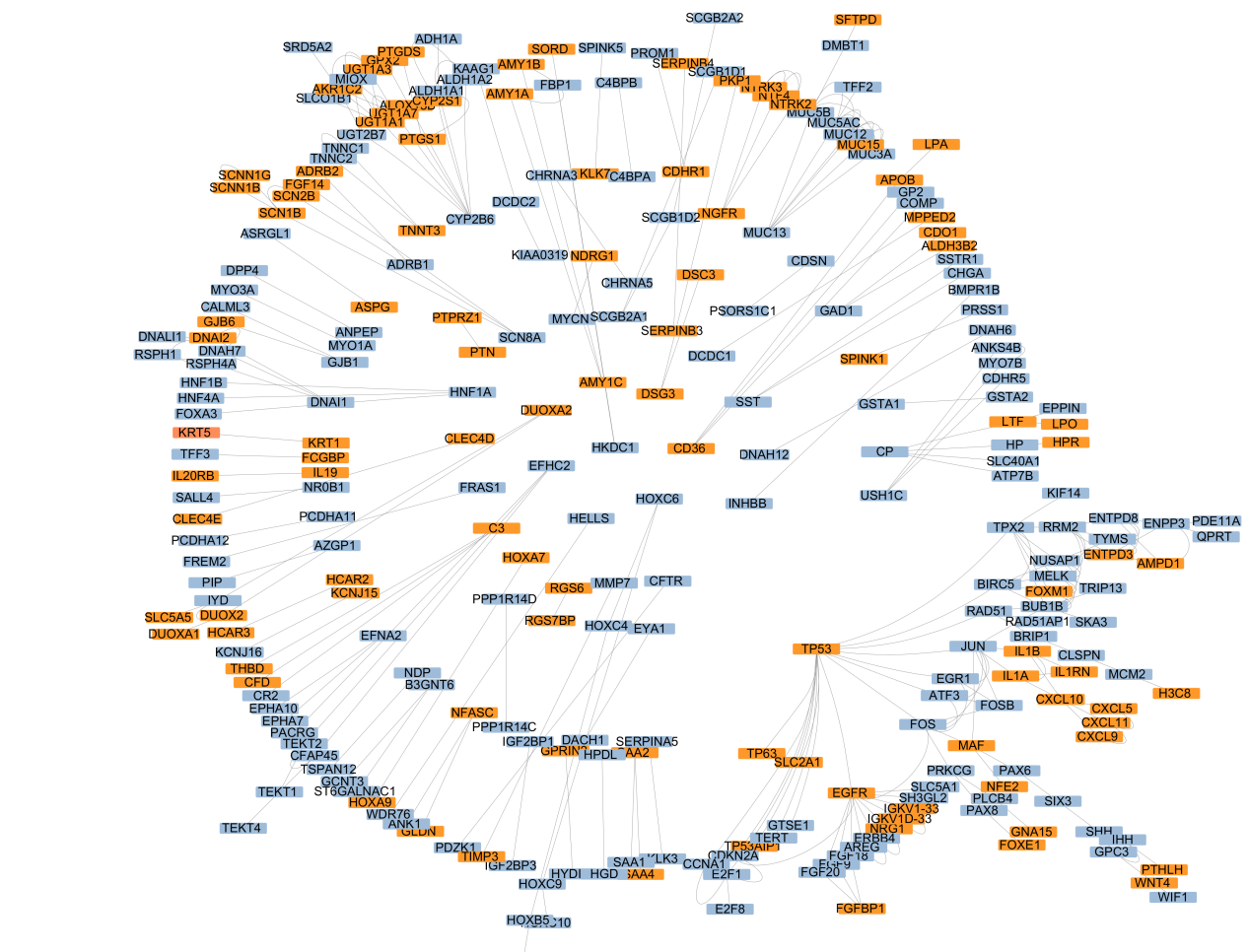
**

Fig:Protein-protein interaction (PPI) network construction.
