# Supplementary figures and images for: Machine learning modeling and analysis of prognostic hub genes in cervical adenocarcinoma: a multi target therapy for enhancement in immunosurveillance
Source: Discov Oncol. 2025 Jul 13;16:1326. doi: 10.1007/s12672-025-02834-3 (PMC12256379; doi:10.1007/s12672-025-02834-3)

**Lollipop alterations among individual’s hub genes**

| **TP53** | **MYC** |
| --- | --- |
| **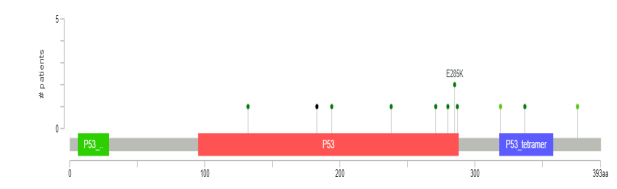** | **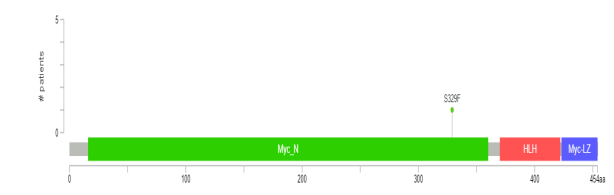** |
| **MUC5B** | **KRT5** |
| **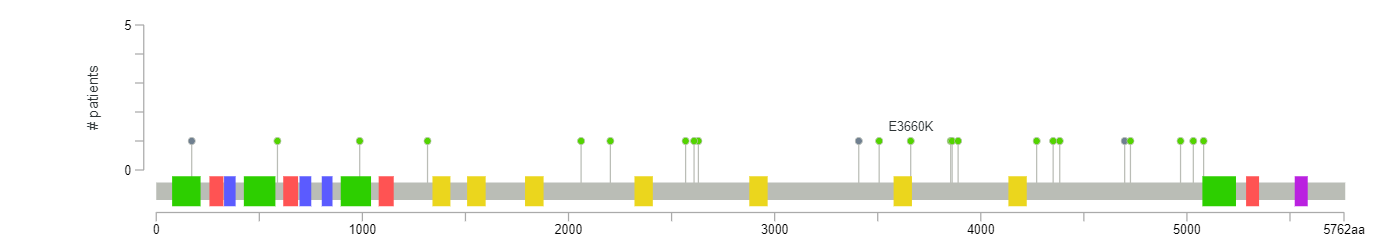** | **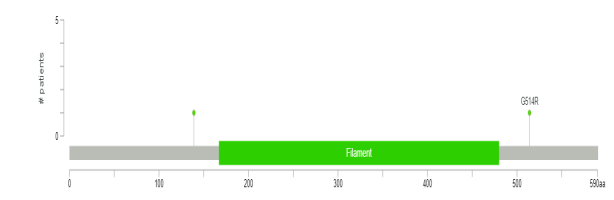** |
| **IL1B** | **CDKN2A** |
| **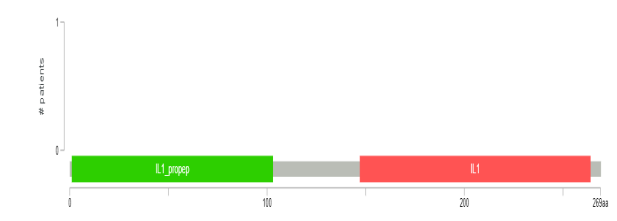** | **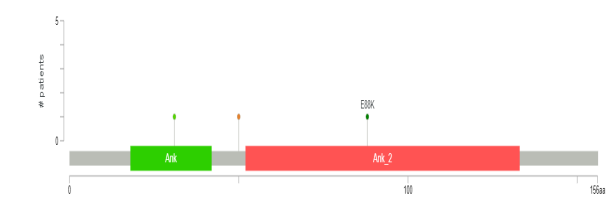** |
| **CCR9** | **CALML3** |
| **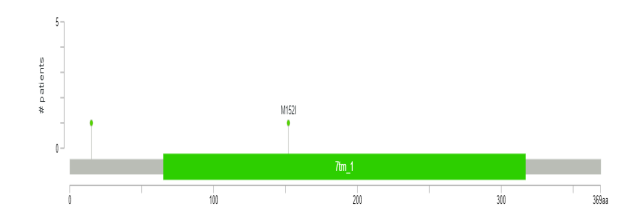** | **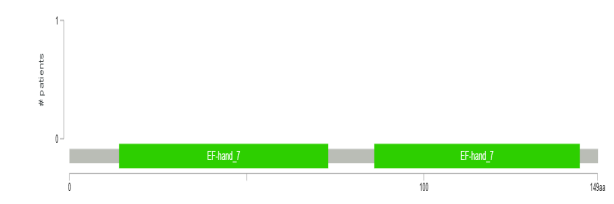** |
| **BUB1B** | **BIRC5** |
| **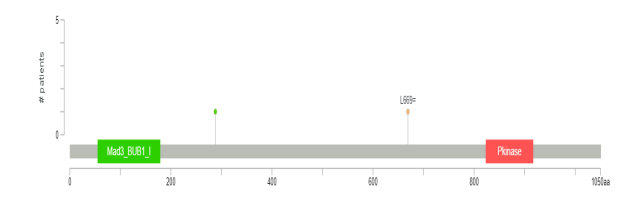** | **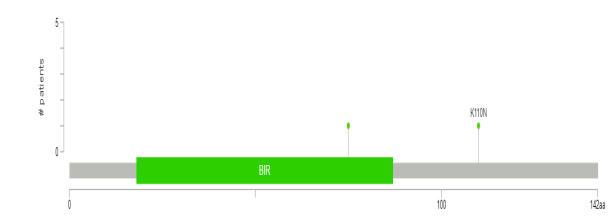** |

Supplement: Supplementary file 4 — Supplementary material 4 [file 12672_2025_2834_MOESM4_ESM.docx]
