# Supplementary material for: Machine learning modeling and analysis of prognostic hub genes in cervical adenocarcinoma: a multi target therapy for enhancement in immunosurveillance
Source: Discov Oncol. 2025 Jul 13;16:1326. doi: 10.1007/s12672-025-02834-3 (PMC12256379; doi:10.1007/s12672-025-02834-3)
Supplement: Supplementary file 5 — Supplementary material 5 [file 12672_2025_2834_MOESM5_ESM.docx]

**Tumor immune Infiltration Analysis of hub genes**

| Gene Names | CD8+T cell | B Cell | Macrophages | Neurophils |
| --- | --- | --- | --- | --- |
| 1.CDKN2A | 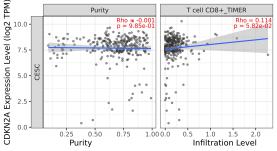 | 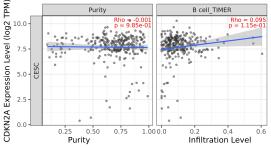 | 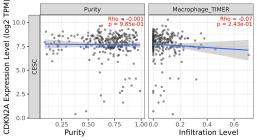 | 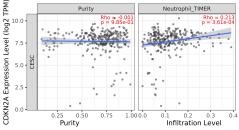 |
| 2.TP53 | 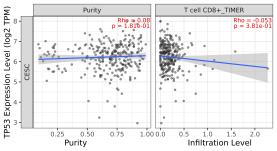 | 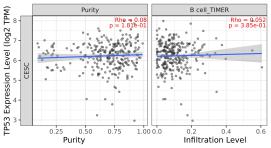 | 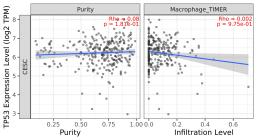 | 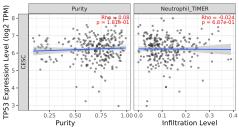 |
| 3.CCR9 | 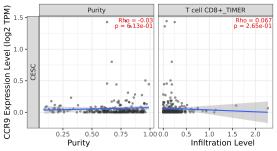 | 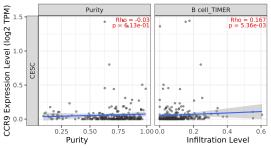 | 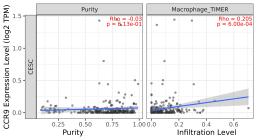 | 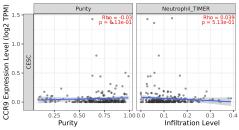 |
| 4.BUB1B | 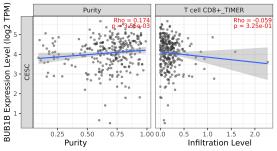 | 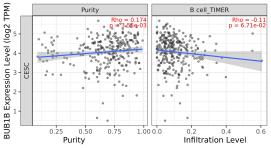 | 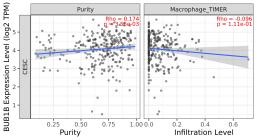 | 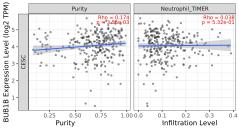 |
| 5.BIRC5 | 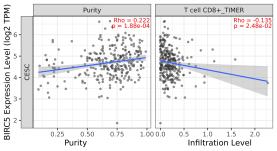 | 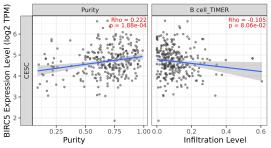 | 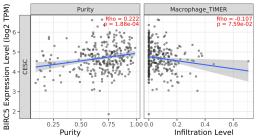 | 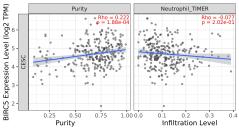 |
| 6.KRT5 | 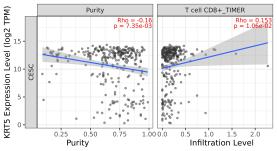 | 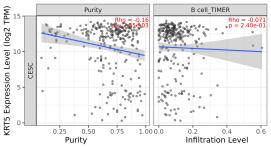 | 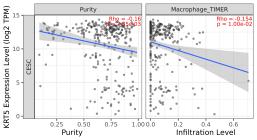 | 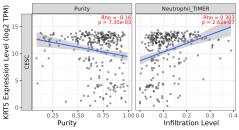 |
| 7.MUC5B | 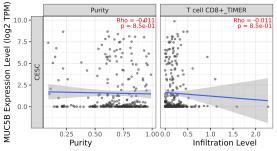 | 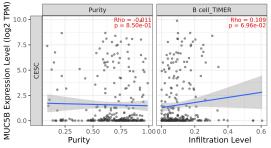 | 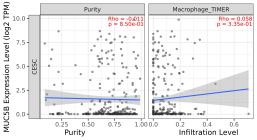 | 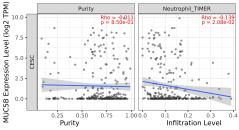 |
| 8.MYC | 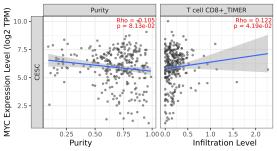 | 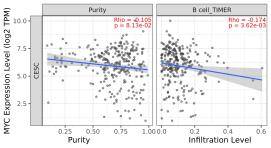 | 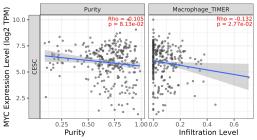 | 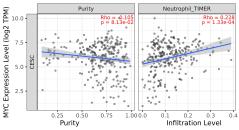 |
| 9.CALML3 | 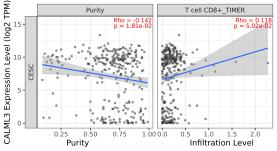 | 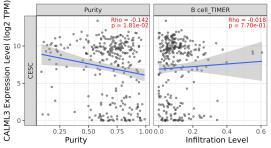 | 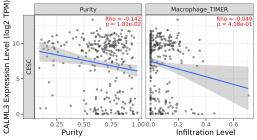 | 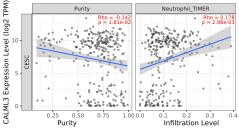 |
| 10.IL1B | 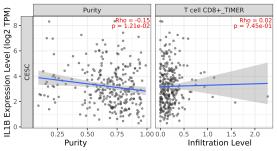 | 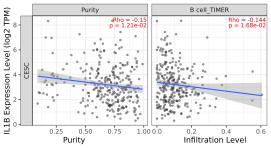 | 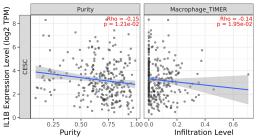 | 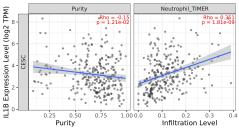 |
