# Supplementary material for: Machine learning modeling and analysis of prognostic hub genes in cervical adenocarcinoma: a multi target therapy for enhancement in immunosurveillance
Source: Discov Oncol. 2025 Jul 13;16:1326. doi: 10.1007/s12672-025-02834-3 (PMC12256379; doi:10.1007/s12672-025-02834-3)
Supplement: Supplementary file 6 — Supplementary material 6 [file 12672_2025_2834_MOESM6_ESM.docx]

**Drugs Targeting Corresponding Genes:**

**
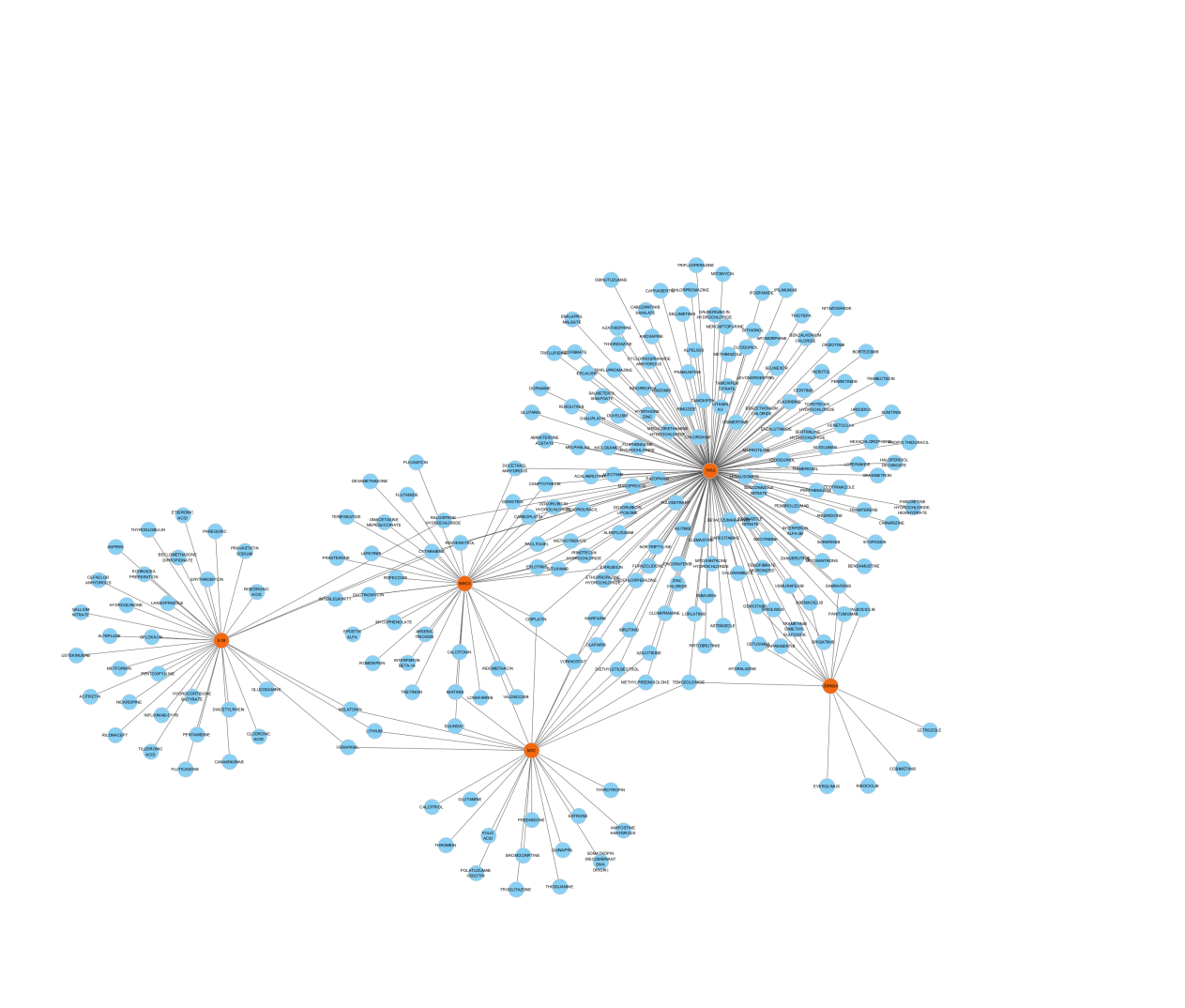
**

**Fig:** shows drugs targeting corresponding genes in which orange circle shows hub genes and blue circle shows drugs.
