# Supplementary material for: Machine learning modeling and analysis of prognostic hub genes in cervical adenocarcinoma: a multi target therapy for enhancement in immunosurveillance
Source: Discov Oncol. 2025 Jul 13;16:1326. doi: 10.1007/s12672-025-02834-3 (PMC12256379; doi:10.1007/s12672-025-02834-3)

**Molecular Dynamics Simulation**

The results depicted as graphs represents stability in terms of deformability and Bfactor. The peaks shows deformability of protein residues higher hinges means higher deformability whereas Bfactor compute comparison between PDB and NMA files the gray color shows PDB file while orange shows NMA and the fluctuation is depicted by color differentiation hinges among them as illustarted in (**Fig 7a**).The eigenvalue graph with purple hinges represented in (**Fig 7b)** measures rigidity of motion which has inverse association with the energy required to distort the structure. While variance graph with green bars as depicted in (**Fig 7c**) calculate normal mode and has inverse correlation with eigenvalue.The covariance matrix graph on basis of Cα Cartesian coordinates representets linkage among protein residues.The red color in graph shows associated residues, white shows unassociated while blue shows anti parallel residues among docked complex **(Fig 7d**).


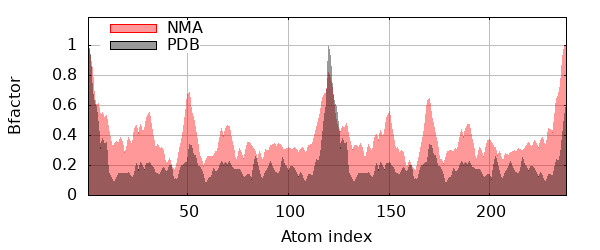

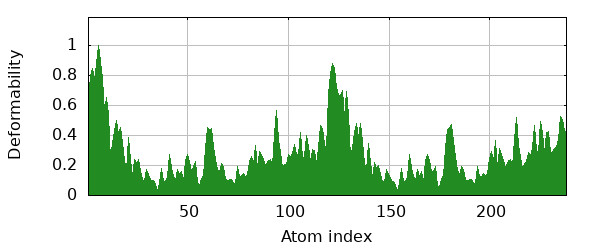


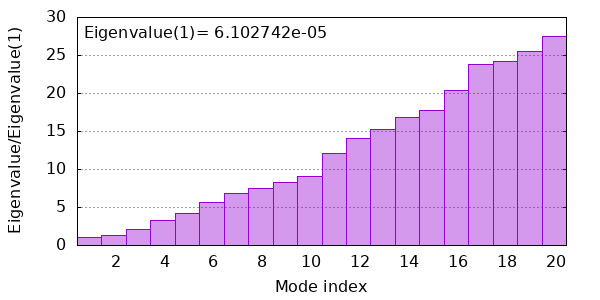

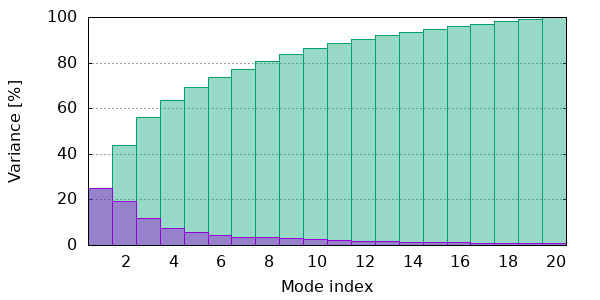


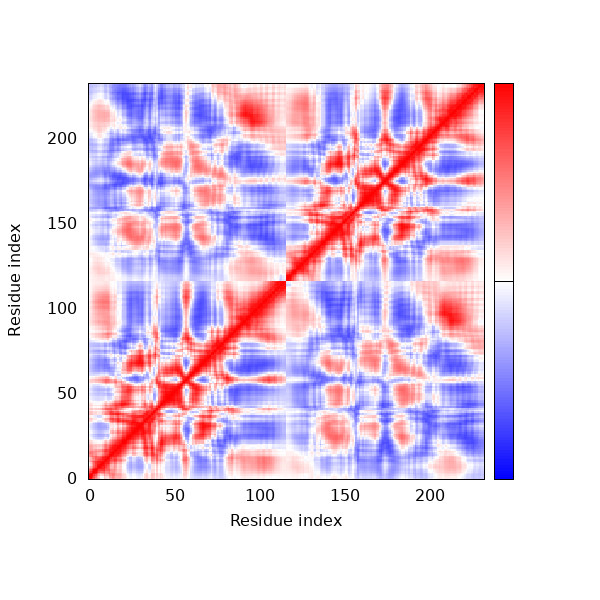

Supplement: Supplementary file 7 — Supplementary material 7 [file 12672_2025_2834_MOESM7_ESM.docx]
